# Multimorbidity patterns and mental health in late life: a systematic review of longitudinal studies

**DOI:** 10.1007/s41999-025-01370-1

**Published:** 2025-12-05

**Authors:** Francesco Palmese, Francesca Remelli, Serhiy Dekhtyar, Giulia Grande, Alessandra Marengoni, Amaia Calderón-Larrañaga, Marco Domenicali, Stefano Volpato, Davide Liborio Vetrano, Federico Triolo

**Affiliations:** 1 Aging Research Center, Department of Neurobiology, Care Sciences and Society, Karolinska Institutet and Stockholm University, Tomtebodavägen 18 A, 171 65 Solna, Stockholm, Sweden; 2 Department of Medical and Surgical Sciences, Alma Mater Studiorum University of Bologna, Ravenna Campus, Ravenna, Italy; 3 Department of Primary Health Care, Internal Medicine Unit Addressed to Frailty and Aging, “S. Maria Delle Croci” Hospital, AUSL Romagna, Ravenna, Italy; 4 Department of Medical Sciences, University of Ferrara, Ferrara, Italy; 5 Stockholm Gerontology Research Center, Stockholm, Sweden; 6 Department of Clinical and Experimental Sciences, University of Brescia, Brescia, Italy; 7 Department of Psychiatry, Amsterdam UMC, Vrije Universiteit, Amsterdam, The Netherlands

**Keywords:** Multimorbidity patterns, Depression, Dementia, Anxiety, Suicide, Epidemiology of Aging, Personalized medicine

## Abstract

**Introduction:**

Several chronic disease combinations (i.e., multimorbidity [MM] patterns) have been linked to poor mental health. This systematic review aimed to synthesize evidence on the longitudinal association between MM patterns and several mental health conditions in later life, including depression, anxiety, suicidality, cognitive decline, and dementia.

**Methods:**

Following PRISMA guidelines (PROSPERO: CRD42024537617), we included longitudinal studies of middle-to-older individuals (45 +) that examined baseline MM patterns and the incidence or trajectories of depression, anxiety, dementia, cognitive decline, or suicidality. The search was conducted in MEDLINE and Web of Science from inception to March 2024, and involved independent screening and quality assessment using a modified Newcastle–Ottawa Scale.

**Results:**

From 13,771 retrieved records, 17 studies were included, ranging from 1209 to 447,888 participants. Fourteen studies were population-based, with follow-ups between 2 and 16 years. Most studies investigated depression (n = 7) and dementia/cognitive decline (n = 9). MM pattern definitions varied, most often relying on data-driven methods (e.g., latent class analysis) and encompassing different numbers and types of diseases. Methodological quality was high across studies. MM patterns featuring cardiometabolic diseases were associated with higher risk of depression, anxiety, cognitive decline, and dementia. Patterns characterized by musculoskeletal, gastrointestinal, and pain-related conditions also showed associations with depression and anxiety. Two studies examined suicidality, with greater multimorbidity burden linked to increased suicidal ideation.

**Conclusion:**

MM patterns linked to higher clinical complexity present poorer mental health trajectories. Validation of MM patterns within and across populations is key for identifying older adults with complex health profiles who may benefit from targeted care strategies.

**Supplementary Information:**

The online version contains supplementary material available at 10.1007/s41999-025-01370-1.

## Key summary points

**Aim:**

To synthesize evidence on the longitudinal association between multimorbidity patterns and several mental health conditions in late life.

**Findings:**

Multimorbidity patterns featuring cardiometabolic diseases were consistently associated with higher risk of depression, anxiety, cognitive decline, and dementia. Patterns characterized by musculoskeletal, gastrointestinal, and pain-related conditions also showed associations with depression and anxiety.

**Message:**

Multimorbidity patterns associated with greater clinical complexity are linked to poorer mental health trajectories in late life.

**Supplementary Information:**

The online version contains supplementary material available at 10.1007/s41999-025-01370-1.

## Introduction

Multimorbidity (MM), the co-occurrence of multiple chronic diseases, is a prevalent condition linked to accelerated aging [1, 2]. When diseases co-occur, they can synergistically increase the risk of disability and frailty, but also of poorer mental health [3–7]. Although most traditional frameworks have focused almost exclusively on physical conditions, growing evidence shows that older adults living with both physical and mental health disorders face particularly elevated risks of functional decline, reduced quality of life, and premature mortality [8–10]. This has led to increasing support for more inclusive definitions of multimorbidity that explicitly incorporate mental health conditions [11, 12]. Embracing this integrated perspective is essential to capturing the complexity of health during aging and guiding person-centered models of care, particularly for mental health [13].

Multimorbidity patterns refer to combinations of chronic conditions that co-occur more frequently than expected by chance [14, 15]. These patterns reflect shared risk factors, including genetic predisposition, lifestyle behaviours, and socioeconomic conditions, as well as age-related physiological changes reflective of biological aging [16]. MM patterns have been differentially associated with a range of adverse outcomes, including disability, unplanned hospitalizations, institutionalization, and mortality [17–20]. As such, they offer a more nuanced way of characterizing health complexity in older adults than simple disease counts. Recognizing MM patterns enables clinicians and researchers to identify more homogeneous subgroups of older individuals with biologically and clinically comparable health profiles [21, 22]. Importantly, understanding MM patterns can facilitate a more targeted study of etiological mechanisms, highlight potential therapeutic or preventive interventions, and refine risk stratification approaches [23].

A growing body of research has explored the association between MM patterns and the development of mental health disorders in older adults. Yet, most studies included in a recent review on the topic have relied on cross-sectional designs [24]. Longitudinal designs are particularly valuable in this context, as they enable to assess whether specific MM patterns precede mental health disorders, clarifying potential causal pathways and helping to identify targets for early intervention. Furthermore, while depression and dementia have received considerable attention [5, 24], other burdensome psychiatric conditions, such as anxiety and suicidality, have remained relatively underexplored despite their high prevalence, frequent co-occurrence in older populations, and high clinical relevance [25, 26]. Evaluating whether specific MM patterns are associated with increased risk of developing these conditions may help identify vulnerable individuals and inform more tailored and effective interventions [1, 27].

In this systematic review, we aimed to summarize the evidence on the longitudinal association between MM patterns and the development of mental health disorders (i.e., depression, anxiety, suicidality, cognitive decline, and dementia) in later life. Although multimorbidity and mental health conditions are likely to be bidirectional, our primary focus was on the association between MM patterns and later mental health consequences, as this direction has garnered the most empirical study to date.

## Methods

This review was conducted and reported in accordance with the Preferred Reporting Items for Systematic Reviews and Meta-Analyses (PRISMA) 2020 guideline (Supplementary Table 1) [28]. The study protocol was registered in PROSPERO (registration number: CRD42024537617). Although subgroup analyses and pooled quantitative estimates were prespecified in the review protocol, these were not conducted due to substantial and unresolvable heterogeneity in study designs, populations, outcome definitions, and analytical approaches. Instead, findings were synthesized narratively.

### Eligibility criteria

We systematically reviewed original studies that investigated the association between multimorbidity patterns as measured in mid or late life and the development of several mental health conditions. Conference abstracts, conference proceedings, non-peer-reviewed reports, dissertations, theses, books, research protocols, and editorials were excluded. Reports in languages other than English were excluded.

Studies were screened according to the following inclusion criteria: (1) participants aged 45 years or older to include middle-to-older individuals; (2) identification of MM patterns, either through theory- (e.g., a priori definitions by bodily systems such as cardiovascular or metabolic patterns) or data-driven approaches (e.g., based on interdependence statistical methods); (3) longitudinal design to study the following mental health conditions: depression, anxiety, suicidality (i.e., from suicidal ideation to death by suicide), cognitive decline, and dementia, modelled in terms of incidence (i.e., development of new cases in individuals without the disorder at baseline) or symptom chronicity over time (i.e., presence of symptoms at follow-up in individuals with the disorder at baseline); and (4) inclusion of several MM patterns contrasted against each other, or relative to individuals without MM. We considered an extensive range of operationalization for the mental health outcomes, including clinical diagnoses (by structured interview or administrative records) and symptom rating scales.

### Information sources and search strategy

Two systematic electronic searches were conducted from inception to March 2024 in the following databases: (1) MEDLINE and PubMed Central (PMC), searched through Ovid, and (2) Web of Science, searched through Clarivate Analytics. The search query combined Medical Subject Headings (MeSH) terms and free-text expressions related to multimorbidity and comorbidity (e.g. [comorbid*], [multimorbid*], [multiple] adj4 [condition*]), patterns of diseases (e.g. [pattern*], [cluster*]), depression (e.g. [depression*], [depressive symptom*]), anxiety (e.g. [anxiety disorder*], [suicid*]), cognitive decline (e.g. [cognitive dysfunction*]), and dementia (e.g. [dementia*], [Alzheimer disease*]). Supplementary Tables 2 and 3 provide full search strategy for each database.

### Selection process

After removing duplicates, each record was screened independently by two reviewers (FP and FR) using Rayyan software [29]. The records judged to meet the screening inclusion criteria by at least one reviewer were evaluated based on full text by three independent reviewers (FP, FR, and FT). The reference lists of the selected studies were individually checked to evaluate any relevant references not included in the selection (i.e., snowball search). Disagreements were resolved through discussion between the three reviewers involved in the selection process.

### Data collection process

A 9-item data extraction tool was initially developed by the senior author (FT) based on predefined variables of interest and pilot-tested on a subset of studies. Two blinded reviewers (FP, and FR) independently extracted information, including study design and characteristics (i.e., first author, year, country, study name, setting, number of participants, mean age, sex, mean follow-up time, disease assessment, MM patterns and their identification method, definition of incidence or chronicity of mental health outcomes, and a summary of main findings). Discrepancies in the extracted information were reviewed and resolved through collegial discussion. Data extraction was performed manually without the use of automation tools or AI software.

### Quality assessment

A modified version of the Newcastle–Ottawa Quality Assessment Form for Cohort Studies was developed to evaluate the quality of the retrieved articles [30]. Modifications reflected the higher quality of studies that reported mental health status at baseline (applicable to studies investigating symptom chronicity), and those providing a detailed description of follow-up time and/or dropout characteristics (score range 0–9, for details see Supplementary Box 1). Each study was rated independently by two reviewers (FP and FR), and differences in evaluations were resolved in discussion with the senior author.

### Synthesis methodology

Given the heterogeneity of the retrieved studies, a narrative synthesis without meta-analysis was performed using harvest plots to visually represent the key findings [31]. These plots provide a standardized visual summary of the distribution and direction of associations between MM patterns and mental health outcomes, along with the sample size of the study from which the association was derived. The harvest plots consisted of six panels, each corresponding to a different outcome: incident depression, chronic depression, incident dementia, cognitive decline, incident anxiety, and chronic anxiety. Due to the specific characteristics of the two retrieved studies on suicidality, they were excluded from the harvest plot, and their results were reported exclusively in the text. Each panel was divided into two sections, displaying studies that reported either a null (i.e., the presence of the multimorbidity pattern was not associated with an increased risk of the outcome) or positive association (i.e., the presence of the multimorbidity pattern was associated with an increased risk of the outcome). Within the plots, each bar represents an association between a specific MM pattern and the respective outcome, with the bar height reflecting the sample size of the study. Additionally, bars were color-coded based on the primary diseases characterizing each MM pattern (see to Supplementary Table 5 for the conversion).

## Results

### Study selection

**Fig. 1:**
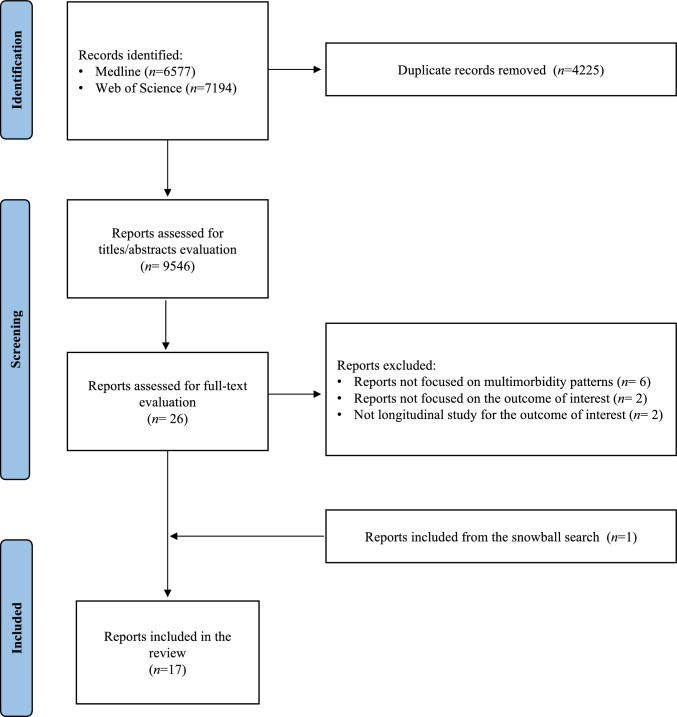
PRISMA flow diagram of the study selection process

We identified 13,771 records, of which 26 studies were retrieved after screening and 17 were selected after full-text evaluation. The study selection process is summarized in Fig. 1.

### Characteristics of the included studies

The characteristics of the 17 included studies are presented in Table 1. Among them, ten studies were conducted in Europe [32–41], four in East Asia [42–45], and three in North America [46–48]. Most studies included population-based samples (n = 14, 82%), while three were performed on clinical cohorts of individuals with depression [33] or with a history of suicidal attempts [47, 48]. The sample sizes ranged from 1209 to 447,888 participants. Although the average age at baseline varied, most studies (n = 14, 82%) focused on participants over 60 years old, while six also included middle-aged individuals [33, 35, 36, 39, 41, 42]. The duration of follow-up varied between 2 and 16 years, but most studies (n = 13, 76%) reported a follow-up over 7 years [32, 34, 37–41, 43–48].

**Table 1:** Characteristics and main findings of the identified studies

First Author, Year	Country, Study name, Study setting	N, Mean age ± SD; Women %	Mean follow-up time ± SD	Disease assessment	Number of diseases included; method to derive multimorbidity patterns	Identified Multimorbidity patterns	Outcome of the study (assessment and operationalization)	Main findings	NOS score
Bendayan et al., 2021	United Kingdom, English Longitudinal Study of Aging (ELSA), Population-based cohort	11,449 Alzheimer’s and dementia- free participants; 65.0 years ± 10.24; female 55%	mean FU NA (FU 14 years)	Physician-diagnosed diseases assessed at wave 1	14 health conditions Data driven patterns derived through latent class analysis	1. Heart Disease/Stroke 2. Asthma/Lung Disease 3. Arthritis/Hypertension 4. Depression/Arthritis 5. Hypertension/Cataracts/Diabetes 6. Psychiatric Problems/Depression 7. Cancer 8. Arthritis/Cataracts	Cognitive performance trajectories (assessed via tests of immediate and delayed recall of 10 common nouns)	Patterns associated at baseline with worse cognitive level compared to the no multimorbidity group: Depression/Arthritis (immediate recall and delayed recall scores) Hypertension/Cataracts/Diabetes (delayed recall score) No Patterns were associated with worse cognitive trajectories	8
Calvin et al., 2022	United Kingdom, UK Biobank, Prospective cohort	206,960 dementia—free participants aged ^3^60 at baseline; 64.1 years ± 2.8; female 52.7%	11.8 years ± 2.2	Participants self-reported medical conditions	42 chronic conditions Data driven patterns derived through latent class analysis	Women: 1. Hypertension, diabetes & coronary heart disease 2. Pain, dyspepsia & depression 3. Cancer 4. Thyroid disorders 5. Pain, osteoporosis & dyspepsia 6. Asthma & COPD 7. Pain & hypertension Men: 1. Hypertension, pain & dyspepsia 2. Pain, dyspepsia & prostate disorders 3. Coronary heart disease, hypertension & stroke 4. Asthma, COPD & psoriasis 5. Diabetes & hypertension 6. Cancer	Incident diagnosis of dementia ascertained using hospital inpatient and death registry records	Patterns associated with the highest dementia in compared to those without multimorbidity: Women Hypertension, diabetes, and coronary heart disease (HR, 2.20 [95% CI, 1.98–2.46]) Pain, osteoporosis, and dyspepsia cluster (HR, 2.00 [95% CI, 1.68–2.37]) Men Diabetes and hypertension (HR, 2.24 [95% CI, 1.97–2.55]) and coronary heart disease, hypertension, and stroke (HR, 1.94 [95% CI, 1.71–2.20])	9
Gerrits et al., 2013	The Netherlands, The Netherlands Study of Depression and Anxiety (NESDA), cohort study	1209 participants with either depression anxiety at baseline with 2-year follow-up	2 years	Self-reported diseases assessed with standardized questionnaire by healthcare professional	43 chronic conditions Theory-driven multimorbidity patterns	1. Cardiometabolic 2. Respiratory 3. Musculoskeletal 4. Digestive 5. Neurological 6. Endocrine 7. Cancer	Presence at follow-up of clinical diagnoses of depression and/or anxiety ascertained through CIDI interview (DSM criteria)	Compared to those without the disease pattern, a 2-year chronic course of depression was associated with: Cardiometabolic (OR 1.54 (1.02–2.31)) Respiratory (OR 0.99 (0.63–1.56)) Musculoskeletal (OR 0.99 (0.63–1.56)) Digestive (OR 0.95 (0.64–1.42)) Neurological (0.61 (0.30–1.25)) Endocrine (OR 1.08 (0.47–2.47)) Cancer (OR 1.18 (0.66–2.13))	9
Grande et al., 2021	Sweden, Swedish National Study on Aging and Care in Kungsholmen (SNAC-K), Population-based cohort	2478 dementia- free participants; 75.0 years ± 10.4; female 64.3%	8.4 years ± 3.9	Integration of physical examination, medical history, self-reported information, and/or proxy interview	Chronic disease coded according to the ICD-10 and classified into 60 chronic disease categories 35 categories were included in the cluster analysis Data driven patterns derived through fuzzy c-means cluster analysis	1. Neuropsychiatric 2. Cardiovascular 3. Sensory impairment/cancer 4. Respiratory/metabolic/musculoskeletal 5. Unspecific	Incident diagnosis of dementia made in accordance with the DSM-IV criteria	Patterns associated with dementia compared to those with unspecific multimorbidity: Neuropsychiatric (HR 1.66; 95% CI 1.13, 2.42) Cardiovascular (HR 1.61; 95% CI 1.17, 2.29) Sensory impairment/cancer (HR 1.32; 95% CI 1.10, 1.71)	9
Ho et al., 2023	Taiwan, Taiwan Longitudinal Survey on Aging (TLSA), Population-based cohort	1975 depression- free participants; 62.1 years ± 7.6; female 48.7%	mean FU NA (FU 16 years)	Participants self-reported medical conditions	12 chronic conditions Data driven patterns derived through latent class analysis	1. Cardiometabolic 2. Arthritis-cataract 3. Multimorbidity 4. Relatively healthy	Incident diagnosis of depression (assessed by using the 10-item short form of the Center for Epidemiologic Studies Depression Scale CES-D)	Compared to those in the relatively healthy group, those in the Cardiometabolic (OR 1.33, CI 0.745–2.375) and in the Arthritis-cataract (OR 1.23, CI 0.704–2.147) were associated with incident depression Among those with poor social participation, incident depression was associated only with the arthritis-cataract pattern (OR 2.218 [95% CI, 1.030–4.778])	8
Hsu et al., 2013	Taiwan, Taiwan Longitudinal Survey on Aging (TLSA), Population-based cohort	4764 depression- free participants; 65.7 years ± 9.2; female 46.2%	mean FU NA (FU 11 years)	Participants self-reported medical conditions	6 self-reported chronic diseases (out of 10) that were associated with the outcome were evaluated Theory-driven patterns based on disease combinations	1. Chronic respiratory disease 2. Cardiovascular disease 3. Gastrointestinal disease 4. Cancer 5. CRD + CVD 6. CRD + GI 7. CRD + cancer 8. CVD + GI 9. CVD + cancer 10. GI + cancer	Depression trajectories (assessed by using the 10-item short form of the Center for Epidemiologic Studies Depression Scale CES-D)	Significant negative effect on the change of depressive symptoms over time: CRD + CVD: − 0.279 CRD + GI: − 0.386	8
Hu et al., 2022	United Kingdom, UK Biobank, Prospective cohort	245,483 dementia- free participants aged ^3^55 at baseline; 62.32 years ± 4.08; female 53.16%	median 9.26 years IQR 7.15–10.78	Hospital inpatient diagnoses	Chronic disease coded according to the ICD-10 and classified into 59 long term conditions 29 categories were included in the cluster analysis Data driven patterns derived through fuzzy c-means cluster analysis	1. Obesity/other disorders 2. Cardio-cerebrovascular/respiratory/metabolic/musculoskeletal/depressive disorders 3. Tumor/genitourinary/digestive disorders	Incident diagnosis of dementia (diagnosed and classified according to the ICD9-10 codes and Read 2–3 codes)	Compared to non-multimorbid participants, those with the cardio-cerebrovascular/respiratory/metabolic/musculoskeletal/depressive multimorbidity were 1.46, 1.28, and 2.50 times more likely to develop all-cause dementia (HR = 1.46, 95% CI = 1.28–1.67), Alzheimer’s disease (HR = 1.28, CI = 1.04–1.58), and vascular dementia (HR = 2.50, CI = 1.90–3.27), respectively Those with tumor/genitourinary/digestive disorders had a 11% higher hazard of Alzheimer’s disease (HR = 1.11, CI = 1.00–1.24) and a 73% elevated risk of vascular dementia (HR = 1.73, CI = 1.37–2.18) compared to those without multimorbidity	9
Li et al., 2023	Europe, Survey of Health, Ageing and Retirement in Europe (SHARE), population based-cohort	16,153 cognitive diseases and Parkinson disease-free participants 50 + ; 65.0 years (IQR 58–85); female 56.7%	Mean FU NA (FU 7 years)	Self-reported information through computer‐assisted personal interviews	12 health conditions (heart attack, hypertension, high blood cholesterol, stroke, diabetes, chronic lung disease, arthritis, cancer, peptic ulcer, cataracts, hip fracture, femoral fracture, and other fractures) Data driven patterns derived through latent class analysis	1. Cardiometabolic 2. High comorbidity burden 3. Osteoarthrosis 4. Low comorbidity burden	Cognition functions, measured by neuropsychological battery	Compared to those in the low comorbidity burden, all patterns presented accelerated cognitive decline Cardiometabolic: ß − 0.059 (− 0.083, − 0.035) High comorbidity burden: ß − 0.116 (− 0.180, − 0.052) Osteoarthrosis: ß − 0.034 (− 0.066, − 0.002)	9
Khondoker et al., 2023	United Kingdom, UK Biobank, Population-based cohort	447,888 dementia-free participants 40–69 years; 58.0 years (IQR 50.0–63.0); female 54.3%	11.3 years (IQR 10.6–12.0)	Linkage to routinely available national datasets, primary care records, cancer screening data, and disease-specific registers	27 clinically relevant chronic diseases in ageing and dementia research Data driven patterns derived through latent class analysis	1. Mental health 2. Cardiometabolic 3. Inflammatory/autoimmune 4. Cancer-related pathophysiology	Incident dementia	Compared to those without MM: Mental health (HR, 2.12; 95% CI: 1.88, 2.39) Cardiometabolic (HR, 2.02;95% CI: 1.87, 2.19)	9
Morin et al., 2023	United States, Department of Veterans Affairs (VA) national data, clinical cohort	2269 VA individuals (65 +) with history of attempted suicide; 70.6 years ± 6.4; female 2.4%	Mean FU NA (FU 7 years)	ICD-9 codes reported in the records of the National Patient Care Database (NPCD) and of the Centers for Medicare & Medicaid Services (CMS) within 2 years before the last visit before the suicide attempt	10 psychiatric (depression, dysthymia,bipolar disorder, post-traumatic stress disorder, generalized anxiety disorder, alcohol abuse, drug abuse, tobacco dependence, schizophrenia, personality disorder) and 12 medical (myocardial infarction, congestive heart failure, stroke, chronic obstructive pulmonary disease, cancer, dementia, traumatic brain injury, hepatitis C, osteoarthritis, renal disease, chronic pain, sleep disorder) chronic diseases Data driven patterns derived through latent class analysis	1. Depression + Minimal Comorbidity 2. Depression + Medical Comorbidity 3. High Comorbidity	Fatal suicide (National Suicide Data Repository and National Suicide Prevention Applications Network)	Those in the Depression + Minimal Comorbidity group had the highest proportion of fatal attempts (33.5%) while those in the High Comorbidity group had the lowest (13.2%), despite the higher likelihood to report documented prior suicidal ideation (*p* < 0.001)	9
Morin et al., 2019	United States, Department of Veterans Affairs (VA) national data, clinical cohort	2131 patients 65 + visited at a VA healthcare facility in primary care; 74.4 (7.8); female 1.8%	Mean FU NA (FU 3 years)	ICD-9 codes reported in the records of the National Patient Care Database (NPCD) and of the Centers for Medicare & Medicaid Services (CMS) within 2 years before the last visit before the suicide attempt	10 psychiatric (depression, dysthymia, bipolar disorder, post-traumatic stress disorder, generalized anxiety disorder, alcohol abuse, drug abuse, tobacco dependence, schizophrenia, personality disorder) and 12 medical (myocardial infarction, congestive heart failure, stroke, chronic obstructive pulmonary disease, cancer, dementia, traumatic brain injury, hepatitis C, osteoarthritis, renal disease, chronic pain, sleep disorder) chronic diseases Data driven patterns derived through latent class analysis	1. Minimal Comorbidity 2. Chronic Pain-Osteoarthritis 3. Depression-Chronic Pain (22.9%) 4. Depression-Medical Comorbidity 5. High Comorbidity	Suicide attempt (National Suicide Data Repository and National Suicide Prevention Applications Network)	Chronic Pain-Osteoarthritis and the Minimal Comorbidity groups had the higher proportion of fatal suicidal attempt (86.0% and 73.4%, respectively). The High Comorbidity group had the lowest (9.6%) but with the highest rate of previous documented suicidal ideation (*p* < 0.001)	8
Ronaldson et al., 2021	United Kingdom, UK Biobank, Population-based cohort	154,367 participants (median age 57 years, IQR 50–62 years; 56.5% female	mean FU 7.6 years, 0.87 SD	Self-report lifetime diagnoses and linked hospital admission records	36 chronic somatic conditions after excluding psychiatric and non-chronic conditions Data driven patterns derived through exploratory factor analysis	1. Undefined multimorbidity 2. Cardiometabolic, 3. Respiratory 4. Cardio/cerebrovascular 5. Reproductive 6. Pain/gastrointestinal	Depression and anxiety measured at baseline: self-report, Patient Health Questionnaire (PHQ)-2, or diagnosis through electronic hospital records Depression outcome: Patient Health Questionnaire (PHQ)-9 Anxiety outcome: Generalised Anxiety Disorder (GAD)-7	Compared to those without multimorbidity (0–1 disease), most patterns were associated with a higher likelihood of developing depression and anxiety Specifically, the cardio/cerebrovascular (aOR 2.11 95% CI 1.45 to 3.07), the respiratory pattern (aOR = 3.23, 95% CI 2.44 to 4.27), and pain/gastrointestinal pattern (aOR = 2.19, 95% CI 1.92 to 2.50) emerged as the strongest predictors of incident depression For incident anxiety, the strongest predictors were the cardiometabolic (aOR 1.42 95% CI 1.16 to 1.72), the respiratory pattern (aOR = 1.75, 95% CI 1.15 to 2.66), and pain/gastrointestinal pattern (aOR = 1.90, 95% CI 1.62 to 2.23) The same patterns were also the strongest predictors among participants with baseline depression or anxiety	9
Triolo et al., 2024	Sweden, Swedish National Study on Aging and Care in Kungsholmen (SNAC-K), Population-based cohort	2904 dementia- and depression free participants; 73.2 years ± 10.5; female 63.1%	9.6 years ± 4	Integration of clinical examination, medication review, linkage to national health records	60 clinically relevant chronic diseases for older people combining up to 918 ICD codes Data driven patterns derived through latent class analysis among participants with ≥ 2 diseases	1. Unspecific 2. Metabolic 3. Sensory/anaemia 4. Thyroid/musculoskeletal 5. Cardiometabolic	Incident diagnosis of depression (Major or minor depressive episode according to DSM-IV)	Patterns associated with depression compared to those without multimorbidity (< 2 diseases): Sensory/anaemia (HR 1.16; 95% CI: 1.08, 1.24); Thyroid/musculoskeletal (HR, 1.91; 95% CI: 1.03, 3.53): Cardiometabolic (HR, 2.77; 95% CI: 1.40, 5.46) Cardiometabolic pattern also associated with depression in multimorbid subsample (reference Unspecific: HR, 1.71 95% CI: 1.02, 2.84)	9
Valletta et al., 2021	Sweden, Swedish National Study on Aging and Care in Kungsholmen (SNAC-K), Population-based cohort	3112 dementia- free participants; 73.6 years ± 10.7; female 63.4%	Up to 15 years	Integration of physical examination, medical history, self-reported information, and/or proxy interview	Chronic disease coded according to the ICD-10 and classified into 60 chronic disease categories 37 categories were included in the cluster analysis Data driven patterns derived through fuzzy c-means cluster analysis	1. Neuropsychiatric 2. Cardiovascular 3. Sensory impairment/cancer 4. Respiratory/metabolic/musculoskeletal 5. Unspecific	Incident diagnosis of dementia made in accordance with the DSM-IV criteria Incidence of Cognitive impairment, no dementia (CIND), based on a neuropsychological test battery	Patterns associated with progression from CIND to dementia compared to those with unspecific multimorbidity: Cardiovascular: HR 1.70, 95% CI 1.15–2.52 Patterns associated with a lower reversion from CIND to normal cognition compared to those with unspecific multimorbidity: Neuropsychiatric (HR 0.53, 95% CI 0.33–0.85 Sensory impairment/cancer HR 0.60, 95% CI 0.39–0.91)	9
Wister et al., 2023	Canada, Canadian Longitudinal Study of Aging (CLSA), Population-based cohort	18,099; 55% between 65 and 74 years, remaining older; female 51.5%	Not reported (from 2 to 5 years)	Self-reported information on diseases in a structured interview	27 self-reported chronic Theory-driven pattern based on presence of ≥ diseases per system	1. Cardio 2. Osteo 3. Respiratory 4. Cancer 5. Multiple 6. Other	Change in depressive (measured with CESD-10) and anxiety (measured with GAD-7) symptoms	Patterns associated with change in depressive symptoms compared to those without the cluster: Respiratory: 2.76, 1.91, 3.60 Cardio: 1.35, 1.08, 1.63 Osteo: 1.67, 1.38, 1.96 Multiple: 2.09, 1.82, 2.36 Patterns associated with change in anxiety symptoms compared to those without the cluster: Cardio: 0.60, 0.41, 0.79 Cancers: 0.47, 0.28, 0.65 Others: 0.61, 0.45, 0.76	9
Xiong et al., 2023	China, China Health and Retirement Longitudinal Study, Population-based cohort	4923 free of motor-cognitive risk syndrome; 66.5 years ± 5.98; female 49.7%	Up to 4 years	Self-reported information in face‐to‐face computer‐assisted personal interviews	13 chronic diseases Data-driven patterns through latent class analysis	1. Relatively healthy pattern 2. Respiratory 3. Cardiovascular	Incidence of Motoric cognitive risk syndrome, defined as co-presence of cognitive complaints (self-reported) and slow gait speed (1SD below age- and sex-adjusted mean) without dementia or impaired mobility	Compared to those in the relatively healthy pattern: Respiratory pattern: HR 1.31 (0.91–1.92) Cardiovascular pattern: HR 1.57(1.16–2.13)	9
Yao et al., 2020	China, China Health and Retirement Longitudinal Study, community based	10,084 participants, mean age 57.7, 46.7 female	Up to 4 years	Self-reported information recorded by a doctor	List of 15 somatic diseases Data driven patterns derived through exploratory factor analysis	1. Cardio-Metabolic 2. Respiratory 3. Arthritic-Digestive-Visual 4. Hepatic-Renal-Skeletal	10-item Center for Epidemiological Studies Depression Scale (CES-D), ≥ 10 as cut-off for depression	All different multimorbidity patterns showed association with depressive symptoms in non- mutually adjusted analyses, with strongest coefficients for Respiratory (aOR: 1.25, 95% CI 1.17, 1.33) and Arthritic-Digestive-Visual patterns (aOR: 1.29, 95% CI 1.22, 1.37)	9

### Quality assessment

The overall quality of the included studies was high, with scores ranging from eight to nine points on the modified Newcastle–Ottawa Scale. The only source of variation across studies related to the adequacy in the report of the follow-up length. Detailed results by domain are presented in Supplementary Table 4.

### Multimorbidity assessment

The number of diseases included in the analyses varied substantially, ranging from 6 to 60. While most studies investigating the outcomes of depression and anxiety focused on somatic diseases, those examining cognitive outcomes and suicide also included psychiatric conditions (e.g., depressive and stress-related disorders). Data on disease presence were collected through various methods, including self-report or clinician assessment via structured or computer-assisted interviews (n = 9, 52%), electronic health records (n = 4, 23%), or a combination of multiple sources (n = 4, 23%). Three studies identified multimorbidity patterns by classifying diseases according to predefined bodily systems (e.g., cardiovascular, musculoskeletal patterns) [33, 45, 46], while the majority conducted data-driven analyses. Among these, the most commonly used statistical methods were latent class analysis (LCA; n = 9, 53%) [32, 35, 37, 39, 40, 43, 44, 47, 48], cluster analysis (n = 3, 17%) [34, 38, 41], and exploratory factor analysis (n = 2, 12%) [36, 42]. Most studies identified at least three MM patterns, with the cardiometabolic (n = 15, 88%), respiratory (n = 9, 53%), cancer (n = 7, 41%), and neuropsychiatric (n = 6, 35%) ones emerging most frequently.

**Fig. 2:**
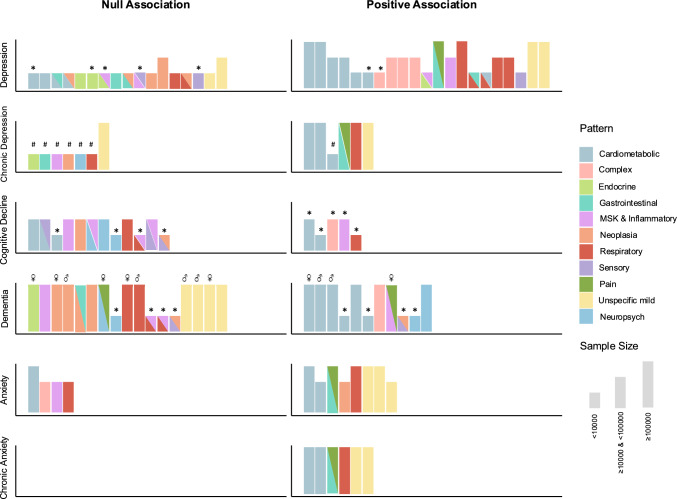
Harvest plot of longitudinal associations between multimorbidity (MM) patterns and different mental health conditions. The horizontal panels represent different outcome, while each bar depicts an null or positive association. The height of the bar indicates the sample size of the study. MSK: musculoskeletal. * Indicates an association between a MM pattern and the outcome using an unspecific MM pattern as the reference group. # Indicates results from [33], where the outcome was the presence of anxiety and/or depression at follow-up in individuals with depression at baseline. ♂ and ♀ indicate that the MM patterns were derived separately for male and female participants [39]

The associations between multimorbidity patterns and the different mental health conditions are summarized in Fig. 2. Across studies, reported effect sizes generally indicated small to moderate associations between specific multimorbidity patterns and adverse mental health outcomes.

### Depression

Seven studies explored the association between baseline multimorbidity patterns and changes in depressive symptomatology. In most studies, depression was assessed using patient-reported measures, such as the Centre for Epidemiological Studies Depression Scale [42, 44–46] or the Patient Health Questionnaire-9 [36]. Conversely, two studies evaluated depression using clinician-assessed measures, that is, the Composite International Diagnostic Interview [33] and the Comprehensive Psychopathological Rating Scale [37]. Three studies focused on depression incidence [37, 42, 44], three on depression trajectories [33, 45, 46], and one on both [36].

Patterns characterized by cardiometabolic/vascular diseases, respiratory diseases, and complex combinations of multiple age-related diseases were most frequently associated with an increased risk of developing depression [36, 37, 42], increasing symptoms over time [45, 46], or chronic depression [33, 36] in comparison with individuals without MM. Notably, cardiometabolic patterns were also associated with increased depression risk in comparison with multimorbid individuals with an unspecific pattern of coexisting diseases (i.e., no over-representation of any of the coexisting diseases) [37]. Other patterns characterized by gastrointestinal, musculoskeletal, and pain-related conditions were associated with an increased risk of depression, despite differences in strength and pattern composition across studies [36, 37, 42, 44–46]. Effect sizes generally ranged between 1.3 and 3.2, with the highest estimates observed for cardiometabolic, respiratory, and musculoskeletal–pain patterns. As shown in Fig. 2, for depression, null findings were predominantly observed in studies with smaller sample sizes.

### Cognitive decline and dementia

Five studies investigated the association between multimorbidity patterns and the incidence of dementia. The diagnosis of dementia was based on ICD [35, 39, 41] or DSM-IV criteria [34, 38]. In all studies, patterns featuring cardiometabolic and vascular diseases were associated with a higher risk of dementia development, relative to the absence of MM [33–36, 38, 39, 41, 46]. Effect sizes generally ranged between 1.3 and 2.5, with the highest estimates observed for cardiometabolic and vascular patterns. Conversely, no statistically significant associations emerged between unspecific patterns and dementia. Additionally, two studies showed associations between neuropsychiatric multimorbidity and dementia onset, relative to those with no MM [35] or with an unspecific pattern [34]. One study explored the association between multimorbidity patterns and dementia subtypes, reporting that, relative to those without MM, multiple coexisting cardiocerebrovascular, respiratory, metabolic, musculoskeletal, and depressive disorders were linked to a higher incidence of both Alzheimer’s disease and Vascular Dementia [41].

One study analyzed multimorbidity patterns in relation to transitions across the cognitive continuum, reporting that individuals with neuropsychiatric and sensory impairment/cancer MM patterns had a lower reversion rate from Cognitive Impairment, No Dementia (CIND) to normal cognition, compared to those with unspecific MM [38]. The same study also found an association between the cardiometabolic pattern and incident dementia among participants with CIND [38]. Last, one study showed that the incidence of the Motoric Cognitive Risk (MCR) syndrome, an at-risk state characterized by cognitive complaints and slow gait speed, was higher in participants with the cardiovascular pattern compared to those in the healthy pattern [43].

Two studies focused on multimorbidity patterns and cognitive trajectories [32, 40]. Compared to participants without MM, only those with depression/arthritis patterns had lower baseline levels in both immediate and delayed recall, while their trajectories over time were not statistically different [32]. Another study showed that individuals with cardiometabolic, osteoarthrosis, or high comorbidity burden patterns experienced faster memory decline compared with those exhibiting a low morbidity pattern [40], as reflected by small but consistent effect estimates (β ≈ − 0.03 to − 0.12).

### Anxiety

Three studies evaluated anxiety [33, 36, 46]. Effect sizes generally ranged between 1.4 and 1.9, with the highest estimates observed for cardiometabolic, respiratory, and gastrointestinal patterns. The cardiometabolic multimorbidity pattern was the one most frequently associated with an increased risk of incident anxiety [36], symptom chronicity [36, 46], and co-presence of anxiety and depression over time in individuals with depression [33]. Other patterns linked to an increased anxiety risk were those characterized by cancer, respiratory, and gastrointestinal patterns [36, 46].

### Suicidality

Two studies examined MM patterns among individuals who had attempted suicide [47, 48]. In both studies, higher morbidity levels were linked to higher suicidal ideation but lower proportion of death by suicide. Patterns characterized by chronic pain-osteoarthritis and minimal morbidity burden presented a higher proportion of deaths by suicide [47, 48].

## Discussion

Despite the considerable heterogeneity among studies, which hampered the possibility of quantitatively synthesizing the findings, this systematic review of 17 studies unveils how multimorbidity patterns are differentially associated with the development of various mental health conditions in late life. Overall, MM patterns characterized by greater clinical complexity, whether due to specific conditions such as cardiometabolic diseases, or the presence of diseases affecting multiple systems, were linked to an increased risk of cognitive decline, dementia, depression, and anxiety. Other co-occurring disease patterns, such as those involving musculoskeletal disorders, gastrointestinal disease or pain-related conditions were also linked to poorer mental health, especially depression and anxiety. Lastly, no associations were observed between unspecific multimorbidity patterns and dementia.

Cardiometabolic multimorbidity patterns, characterized by disease combinations that often include hypertension, diabetes, and cardiovascular diseases, were associated with various mental health outcomes, including dementia, depression, and anxiety. This reinforces previous evidence suggesting that vascular and metabolic mechanisms contribute to the pathophysiology of mental health conditions, particularly dementia and depression [49, 50]. Cardiometabolic burden has been linked to an increased risk of cognitive decline and dementia, potentially due to vascular damage, poorer brain perfusion, and chronic inflammation, ultimately promoting the accumulation of both neurodegenerative and cerebrovascular [51–53]. Similarly, cerebrovascular damage, such as white matter hyperintensities, has been linked to a higher risk of depression, symptom chronification, and poorer response to antidepressant medications [54–57]. Neuroendocrine alterations of the hypothalamic–pituitary–adrenal axis and autonomic nervous system, associated to cardiometabolic diseases, can also affect mood regulation and anxiety [58–60]. Overall, this evidence suggests that poorer cognitive and mental health outcomes in people with patterns of cardiometabolic diseases may be underpinned by specific biological mechanisms.

Conversely, individuals with cardiometabolic multimorbidity are usually characterized by several other age-related diseases, leading to complex disease profiles indicative of advanced biological age [61]. A broad range of biological factors, such as chronic inflammation, oxidative stress, and metabolic dysregulation, are likely contributors to both the development of multiple chronic conditions and the deterioration of mental health, as framed by the geroscience hypothesis [62–64]. As such, common underlying pathways shared by chronic diseases may explain why other MM patterns, such as those characterized by respiratory diseases, cancer, and musculoskeletal disorders, were also linked to adverse mental health outcomes, although with lower strength. Further, other care-related factors may account for why individuals with complex disease profiles are more vulnerable to poorer mental health. Managing MM introduces unique acute and chronic stressors, including receiving new diagnoses, undergoing medical procedures, facing uncertainty, navigating care systems, handling polypharmacy, and dealing with symptoms and disabilities, all of which can induce significant distress [65]. These challenges can exceed an individual's coping resources, which may already be diminished in complex multimorbid patients, further contributing to poorer cognitive and mental health [66]. Factors often present in persons affected by multimorbidity, such as lack of financial and social support and isolation, further hinder effective coping mechanisms, increasing vulnerability to mental health conditions [3]. This underscores the importance of identifying older people with complex disease profiles to implement tailored clinical and public health strategies to effectively prevent poor mental health outcomes.

Notably, studies reporting on unspecific multimorbidity patterns, i.e. lacking overrepresented diseases, found no association with dementia, although associations with other outcomes, particularly anxiety, were more frequently observed. This suggests that the composition of disease patterns, rather than the mere co-occurrence of conditions, may be more relevant for dementia risk, likely reflecting the role of cumulative biological burden. In contrast, the links with anxiety may be driven by psychological mechanisms, such as difficulties coping with illness or navigating complex care needs. These findings underscore the importance of identifying clinically meaningful disease patterns and support the need for more targeted, hypothesis-driven research to better understand the mental health implications of multimorbidity in older people.

### Conceptual and methodological considerations

#### Multimorbidity

A key challenge in interpreting these results lies in how MM patterns were defined and extracted across studies. Various statistical methods were employed, with most studies using latent class analysis. However, there is still no consensus on how to define and operationalize these patterns in terms of the minimum set of diseases required, the populations to which these methods should be applied, the optimal statistical methods for information extraction, or the sample size needed to assure stable results [15]. This lack of standardization hinders comparability across studies and the quantitative synthesis of the findings. Recently, considerable efforts have been made to standardize the definition of MM, including recommendations on core disease lists and the selection of MM operationalizations (e.g., disease counts, weighted indices, MM patterns) according to study objectives (e.g., estimating prevalence, predicting outcomes, examining disease interactions), as well as to develop core outcome sets relevant to individuals with MM (e.g., quality of life and mental health outcomes) [11, 67]. Similarly, establishing a consensus on MM pattern definitions and analytical frameworks is critical for strengthening clinical and public health implications of this research field.

Notably, most studies used individuals without MM as a reference group, although these represent a small share of old-age populations, with multimorbidity prevalence reaching up to 89% in individuals aged 60 and above [68]. Comparing risks among multimorbid individuals with different levels of clinical complexity may be more appropriate within older study populations. Studies have suggested the existence of unspecific multimorbidity patterns including mostly cardiovascular risk factors that can evolve into more severe and complex forms, usually observed in younger seniors and characterized by lower functional impairment [15, 22]. Given the likely progression from milder to more complex forms or MM over time [22], recognizing milder phenotypes as a window of opportunity for preventive actions, both of mental health as well as functional outcomes, is essential. This also emphasizes the importance of incorporating longitudinal designs when studying MM patterns.

#### Mental health outcomes

Studies also presented high variability in how mental health outcomes were measured, posing added challenges for interpreting the results. Most investigations on depression and anxiety employed self-assessed symptom rating scales, which, while valuable for capturing subjective experiences, do not reflect clinical diagnoses and may entail reporting biases. This underscores the need for further studies utilizing clinical diagnostic tools to provide more precise and clinically based assessments of cognitive and psychiatric conditions. Furthermore, most studies focused on a single neuropsychiatric outcome and/or assessed it with few repeated measurements, which may provide a simplified view of mental health in late life [69, 70].

Despite the clinical relevance of anxiety and suicidality, few published studies specifically considered these outcomes. Both anxiety symptoms and suicidal thoughts are common in old age, especially among people living with MM [36, 69, 71]. While anxiety can be easily measured and implemented in future studies, investigating suicidal attempt and deaths by suicide present with specific challenges in terms of measurement, case identification, and study design [72–74]. Nonetheless, suicidal rates are highest among people aged 70 + , underscoring the importance of exploring whether certain MM patterns, alongside broader psychological, social, and environmental factors, contribute to an increased risk of suicide in later life. [26, 75]. Last, most of the studies were conducted in population-based settings and were designed to primarily investigate the incidence of dementia and depression. While this represents a merit from a public health standpoint, evidence from clinical populations is equally valuable to understand how MM patterns influence the chronicity of poor mental health, as well as other conditions such as delirium, particularly in older adults with complex health profiles [13, 76].

### Care implications

Recognizing specific MM patterns associated with adverse mental health outcomes may have significant clinical implications. In particular, it could enable healthcare providers to target appropriate patients with screening tools for cognitive and psychiatric symptoms, consider early tailored interventions that address unique clinical needs, and improve prognosis. Yet, implementing this knowledge in clinical settings remains a challenge. Increased accessibility of electronic health records could facilitate the identification of MM patterns in primary care [15], where collaborative care models involving multidisciplinary teams have been developed and shown to be effective, especially for patients with depression and somatic conditions [77, 78]. Such interventions could enhance patient outcomes by providing comprehensive management of coexisting conditions [79], which in turn could help transition to more person-centered medical and social care through clinical phenotyping based on MM patterns. This is especially important for older individuals with cognitive, psychiatric and somatic comorbidities, who often receive fragmented and sub-optimal care and are at higher risk of disability, placing additional strain on caregivers. Ensuring comprehensive, well-coordinated treatment strategies could not only improve outcomes for these patients but also alleviate the caregiving burden.

### Strengths and limitations

The strengths of this review include the systematic approach, a comprehensive search strategy, pre-registered protocol, and a tailored quality assessment of the included studies. However, some limitations need to be considered. First, limiting the search to two databases may have introduced selection bias. Second, the exclusion of grey literature may have introduced publication bias by omitting relevant unpublished studies. Third, this review was limited to studies published in English, which may have introduced language bias. However, an additional unrestricted search identified only a small proportion of non-English publications, indicating that the potential impact of this limitation is likely minimal. Fourth, generalizability of our findings is potentially limited because the studies included were predominantly conducted in high- or upper-middle-income countries. Fifth, the considerable heterogeneity across studies precluded the assessment of the certainty of evidence regarding the associations. Lastly, our search strategy might not have captured all relevant articles on the clustering of chronic conditions since some studies may not have explicitly used 'multimorbidity' or related terms in their titles, abstracts, or MeSH headings.

## Conclusion

In conclusion, this review suggests a positive and consistent association between complex multimorbidity patterns, mostly characterized by cardiometabolic and musculoskeletal diseases, and adverse mental health conditions such as dementia, cognitive decline, depression, anxiety in older adults. In particular, cardiometabolic multimorbidity patterns may help identify individuals at risk of poor mental health who could benefit from targeted interventions. Future research should focus on standardizing methods for deriving MM patterns, utilizing clinical diagnostic tools for cognitive and psychiatric assessments, and prioritizing the implementation of research findings across care settings. These efforts have the potential to enhance the quality of life of individuals affected by multimorbidity and coexisting mental health conditions.

## Supplementary Information

Below is the link to the electronic supplementary material.

Supplementary file1 (DOCX 81 KB)

## Data Availability

Not applicable as no data have been analysed.
