# Supplementary material for: Multimorbidity patterns and mental health in late life: a systematic review of longitudinal studies
Source: Eur Geriatr Med. 2025 Dec 5;17(2):465–87. doi: 10.1007/s41999-025-01370-1 (PMC13109113; doi:10.1007/s41999-025-01370-1)
Supplement: Supplementary file 1 — Supplementary file1 (DOCX 81 KB) [file 41999_2025_1370_MOESM1_ESM.docx]

Supplementary Table 1. Preferred Reporting Items for Systematic Reviews and Meta-Analyses (PRISMA) 2020 checklist. 2

Supplementary Table 2. Search strategy in MEDLINE. 5

Supplementary Table 3. Search strategy in Web of Science. 7

Supplementary Table 4. Newcastle-Ottawa Quality Assessment Scale (adapted for cohort studies). 8

Supplementary Table 5. Quality assessment of the included studies according to the Newcastle-Ottawa Quality Assessment Scale. 9

Supplementary Table 6. Conversion of colours coding charactering diseases for each multimorbidity patterns presented in the harvest plot (Figure 2). 11

# **Supplementary Table 1.** Preferred Reporting Items for Systematic Reviews and Meta-Analyses (PRISMA) 2020 checklist.

| **Section and Topic** | **Item #** | **Checklist item** | **Location where item is reported** |
| --- | --- | --- | --- |
| **TITLE** | | |  |
| Title | 1 | Identify the report as a systematic review. | Page 1 |
| **ABSTRACT** | | |  |
| Abstract | 2 | See the PRISMA 2020 for Abstracts checklist. | Page 2 |
| **INTRODUCTION** | | |  |
| Rationale | 3 | Describe the rationale for the review in the context of existing knowledge. | Page 3 |
| Objectives | 4 | Provide an explicit statement of the objective(s) or question(s) the review addresses. | Page 3 |
| **METHODS** | | |  |
| Eligibility criteria | 5 | Specify the inclusion and exclusion criteria for the review and how studies were grouped for the syntheses. | Page 4 |
| Information sources | 6 | Specify all databases, registers, websites, organisations, reference lists and other sources searched or consulted to identify studies. Specify the date when each source was last searched or consulted. | Page 4 |
| Search strategy | 7 | Present the full search strategies for all databases, registers and websites, including any filters and limits used. | Page 4 and supplementary materials |
| Selection process | 8 | Specify the methods used to decide whether a study met the inclusion criteria of the review, including how many reviewers screened each record and each report retrieved, whether they worked independently, and if applicable, details of automation tools used in the process. | Page 5 |
| Data collection process | 9 | Specify the methods used to collect data from reports, including how many reviewers collected data from each report, whether they worked independently, any processes for obtaining or confirming data from study investigators, and if applicable, details of automation tools used in the process. | Page 5 |
| Data items | 10a | List and define all outcomes for which data were sought. Specify whether all results that were compatible with each outcome domain in each study were sought (e.g. for all measures, time points, analyses), and if not, the methods used to decide which results to collect. | Page 3 |
|  | 10b | List and define all other variables for which data were sought (e.g. participant and intervention characteristics, funding sources). Describe any assumptions made about any missing or unclear information. | Page 5 |
| Study risk of bias assessment | 11 | Specify the methods used to assess risk of bias in the included studies, including details of the tool(s) used, how many reviewers assessed each study and whether they worked independently, and if applicable, details of automation tools used in the process. | Page 5 |
| Effect measures | 12 | Specify for each outcome the effect measure(s) (e.g. risk ratio, mean difference) used in the synthesis or presentation of results. | N/A |
| Synthesis methods | 13a | Describe the processes used to decide which studies were eligible for each synthesis (e.g. tabulating the study intervention characteristics and comparing against the planned groups for each synthesis (item #5)). | Page 5 |
|  | 13b | Describe any methods required to prepare the data for presentation or synthesis, such as handling of missing summary statistics, or data conversions. | Page 5 |
|  | 13c | Describe any methods used to tabulate or visually display results of individual studies and syntheses. | Page 5 |
|  | 13d | Describe any methods used to synthesize results and provide a rationale for the choice(s). If meta-analysis was performed, describe the model(s), method(s) to identify the presence and extent of statistical heterogeneity, and software package(s) used. | Page 5 |
|  | 13e | Describe any methods used to explore possible causes of heterogeneity among study results (e.g. subgroup analysis, meta-regression). | N/A |
|  | 13f | Describe any sensitivity analyses conducted to assess robustness of the synthesized results. | N/A |
| Reporting bias assessment | 14 | Describe any methods used to assess risk of bias due to missing results in a synthesis (arising from reporting biases). | Page 5 |
| Certainty assessment | 15 | Describe any methods used to assess certainty (or confidence) in the body of evidence for an outcome. | N/A |
| **RESULTS** | | |  |
| Study selection | 16a | Describe the results of the search and selection process, from the number of records identified in the search to the number of studies included in the review, ideally using a flow diagram. | Page 6 |
|  | 16b | Cite studies that might appear to meet the inclusion criteria, but which were excluded, and explain why they were excluded. |  |
| Study characteristics | 17 | Cite each included study and present its characteristics. | Page 6 |
| Risk of bias in studies | 18 | Present assessments of risk of bias for each included study. | Page 6 and supplementary materials |
| Results of individual studies | 19 | For all outcomes, present, for each study: (a) summary statistics for each group (where appropriate) and (b) an effect estimate and its precision (e.g. confidence/credible interval), ideally using structured tables or plots. | Page 6-9 |
| Results of syntheses | 20a | For each synthesis, briefly summarise the characteristics and risk of bias among contributing studies. | Page 6-9 |
|  | 20b | Present results of all statistical syntheses conducted. If meta-analysis was done, present for each the summary estimate and its precision (e.g. confidence/credible interval) and measures of statistical heterogeneity. If comparing groups, describe the direction of the effect. | N/A |
|  | 20c | Present results of all investigations of possible causes of heterogeneity among study results. | N/A |
|  | 20d | Present results of all sensitivity analyses conducted to assess the robustness of the synthesized results. | N/A |
| Reporting biases | 21 | Present assessments of risk of bias due to missing results (arising from reporting biases) for each synthesis assessed. | N/A |
| Certainty of evidence | 22 | Present assessments of certainty (or confidence) in the body of evidence for each outcome assessed. | N/A |
| **DISCUSSION** | | |  |
| Discussion | 23a | Provide a general interpretation of the results in the context of other evidence. | Page 9-12 |
|  | 23b | Discuss any limitations of the evidence included in the review. | Page 12 |
|  | 23c | Discuss any limitations of the review processes used. | Page 12 |
|  | 23d | Discuss implications of the results for practice, policy, and future research. | Page 11-12 |
| **OTHER INFORMATION** | | |  |
| Registration and protocol | 24a | Provide registration information for the review, including register name and registration number, or state that the review was not registered. | Page 4 |
|  | 24b | Indicate where the review protocol can be accessed, or state that a protocol was not prepared. | Page 4 |
|  | 24c | Describe and explain any amendments to information provided at registration or in the protocol. | Page 3 |
| Support | 25 | Describe sources of financial or non-financial support for the review, and the role of the funders or sponsors in the review. | Page 1 |
| Competing interests | 26 | Declare any competing interests of review authors. | Page 1 |
| Availability of data, code and other materials | 27 | Report which of the following are publicly available and where they can be found: template data collection forms; data extracted from included studies; data used for all analyses; analytic code; any other materials used in the review. | N/A |

# **Supplementary Table 2**. Search strategy in MEDLINE.

| Interface | Ovid MEDLINE (R) and Epub Ahead of Print, In- Process, In-Data-Review & Other Non-Indexed Citations, Daily and Versions 1946 to March 14 2024 | |  |
| --- | --- | --- | --- |
| Date of Search | 15-Mar-24 | |  |
| Number of hits | 6577 | |  |
|  | | |  |
| exp/ = exploded MeSH term  / = non exploded MeSH term ti,ab,kf. = title, abstract and author keywords adjx = within x words, regardless of order  * = truncation of word for alternate endings | | |  |
|  |  |  |  |
|  |  |  |  |
|  | | |  |
| # | Searches | Results |  |
| 1 | exp Comorbidity/ | 129257 |  |
| 2 | Multiple chronic conditions/ | 742 |  |
| 3 | (co-morbid* or comorbid* or multi-condition* or multi-morbid* or multicondition* or multimorbid*).ti,ab,kf. | 279033 |  |
| 4 | ((co-exist* or co-occurr* or coexist* or combin* or con-current* or concurrent* or cooccur* or multiple) adj4 (condition* or diagnos* or disease* or health problem* or illness*)).ti,ab,kf. | 150919 |  |
| 5 | 1 or 2 or 3 or 4 | 489094 |  |
| 6 | (pattern* or cluster*).ti,ab,kf. | 1974577 |  |
| 7 | 5 and 6 | 35111 |  |
| 8 | exp Mood Disorders/ | 172874 |  |
| 9 | exp Depression/ | 156101 |  |
| 10 | exp Anxiety Disorders/ | 93510 |  |
| 11 | exp Anxiety/ | 116544 |  |
| 12 | exp Suicide/ | 76635 |  |
| 13 | (depression* or depressive disorder* or depressive episode* or depressive symptom* or anxiety* or suicid*).ti,ab,kf. | 689199 |  |
| 14 | exp Dementia/ | 212830 |  |
| 15 | exp Delirium/ | 13257 |  |
| 16 | exp Cognitive Dysfunction/ | 39684 |  |
| 17 | (dementia* or vascular dementia* or delirium* or alzheimer disease* or cognitive decline* or cognitive impairment* or delirium).ti,ab,kf. | 260806 |  |
| 18 | 8 or 9 or 10 or 11 or 12 or 13 or 14 or 15 or 16 or 17 | 1166874 |  |
| 19 | 7 and 18 | 7555 |  |
| 20 | limit 19 to english language | 7322 |  |
| 21 | (review or congress or clinical conference or comment or editorial or letter).pt. | 5504582 |  |
| 22 | 20 not 21 | 6577 |  |

# **Supplementary Table 3**. Search strategy in Web of Science.

| Interface | Clarivate analytics | | | | | | |  |
| --- | --- | --- | --- | --- | --- | --- | --- | --- |
| Editions | Emerging Sources Citation Index (ESCI), Arts & Humanities Citation Index (A&HCI), Science Citation Index Expanded (SCI-EXPANDED), Social Sciences Citation Index (SSCI) | | | | | | |  |
| Date of Search | 15-Mar-24 | | | | | | |  |
| Number of hits | 7194 | | | | | | |  |
|  | | | | | | | |  |
| TS/Topic = title, abstract, author keywords and Keywords Plus NEAR/x = within x words, regardless of order * = truncation of word for alternate endings the Exact search-function was used for all the searches | | | | | | | |  |
|  |  |  |  |  |  |  |  |  |
|  |  |  |  |  |  |  |  |  |
|  | |  |  |  |  |  |  |  |
| # | | Searches | | | | | Results |  |
| 1 | | TS=("co-morbid*" or comorbid* or "multi-condition*" or "multi-morbid*" or multicondition* or multimorbid*) | | | | | 321652 |  |
| 2 | | TS=(("co-exist*" or "co-occurr*" or coexist* or combin* or "con-current*" or concurrent* or cooccur* or multiple) NEAR/4 (condition* or diagnos* or disease* or "health problem*" or illness*)) | | | | | 218325 |  |
| 3 | | #2 OR #1 | | | | | 528550 |  |
| 4 | | TS=(pattern* or cluster*) | | | | | 4235575 |  |
| 5 | | #3 AND #4 | | | | | 45041 |  |
| 6 | | TS=(mood disorder*or depression* or depressive disorder* or depressive episode* or depressive symptom* or anxiety* or anxiety disorder* or suicid*) | | | | | 638395 |  |
| 7 | | TS=(dementia* or vascular dementia* or delirium* or alzheimer disease* or cognitive dysfunction* cognitive decline* or cognitive impairment*) | | | | | 494339 |  |
| 8 | | #7 OR #6 | | | | | 1100577 |  |
| 9 | | #8 AND #5 | | | | | 8379 |  |
| 10 | | (#9) AND LA=(English) | | | | | 8141 |  |
| 11 | | (#10) NOT DT=(Proceedings Paper OR Meeting Abstract OR Editorial Material OR Review OR Letter) | | | | | 7194 |  |

#

# **Supplementary Box 1**. Modified Newcastle-Ottawa Quality Assessment Scale (adapted for cohort studies).

**Selection**

1) Representativeness of the exposed cohort

a) truly representative *****

b) somewhat representative *****

c) selected group of users

d) no description of the derivation of the cohort

2) Selection of the non exposed cohort

a) drawn from the same community as the exposed cohort *****

b) drawn from a different source

c) no description of the derivation of the non exposed cohort

3) Ascertainment of exposure

a) secure record *****

b) structured interview *****

c) written self report

d) no description

4) Demonstration that outcome of interest was not present at start of study

a) yes *****

b) no

**Comparability**

1) Comparability of cohorts on the basis of the design or analysis

a) study controls for age and sex*****

b) study controls for any additional factor *****

**Outcome**

1) Assessment of outcome

a) independent blind assessment *****

b) record linkage *****

c) self report

d) no description

2) Was follow-up long enough for outcomes to occur

a) yes *****

b) no

3) Adequacy of follow up of cohorts

a) complete follow up - all subjects accounted for *****

b) subjects lost to follow up unlikely to introduce bias - small number lost less than or equal 20% or description provided of those lost suggested no different from those followed*****

c) follow up rate < 80% and no description of those lost

d) no statement

Notes:

- A study can be awarded a maximum of one point for each numbered item within the Selection and Outcome sections;
- A maximum of two point can be given for the Comparability section;
- At the fourth point of the Selection section, for studies investigating chronicity/trajectories we assigned one point if the study reports the mental health status at baseline;
- At the third point of the Outcome section, to assess the adequacy of the follow-up we assigned one point if the study reports the mean (SD) of the follow-up and/or describes the proportion/characteristics of the dropouts.

# **Supplementary Table 4**. Quality assessment of the included studies according to the Newcastle-Ottawa Quality Assessment Scale.

|  | **Selection (maximum of 4 points)** | | | | **Comparability** | **Outcome** | | | **TOTAL** |
| --- | --- | --- | --- | --- | --- | --- | --- | --- | --- |
| **Study** | **1. Representativeness of the exposed cohort** | **2. Selection of the non-exposed cohort** | **3. Ascertainment of exposure** | **4. Demonstration that outcome of interest was not present at start of study** | **1. Comparability of cohort on the basis of the design or analysis (max 2 points)** | **1. Assessment of outcome** | **2. Was follow-up long enough for outcomes to occur** | **3. Adequacy of follow-up of cohorts** | **Total (maximum of 9 points)** |
| Bendayan et al, 2021 | 1 | 1 | 1 | 1 | 2 | 1 | 1 | 0 | 8 |
| Calvin et al, 2022 | 1 | 1 | 1 | 1 | 2 | 1 | 1 | 1 | 9 |
| Gerrits et al, 2013 | 1 | 1 | 1 | 1 | 1 | 1 | 1 | 1 | 9 |
| Grande et al, 2021 | 1 | 1 | 1 | 1 | 2 | 1 | 1 | 1 | 9 |
| Ho et al, 2023 | 1 | 1 | 1 | 1 | 2 | 1 | 1 | 0 | 8 |
| Hsu et al,  2013 | 1 | 1 | 1 | 1 | 2 | 1 | 1 | 0 | 8 |
| Hu et al, 2022 | 1 | 1 | 1 | 1 | 2 | 1 | 1 | 1 | 9 |
| Li et al, 2023 | 1 | 1 | 1 | 1 | 2 | 1 | 1 | 1 | 9 |
| Khondoker et al,  2023 | 1 | 1 | 1 | 1 | 2 | 1 | 1 | 1 | 9 |
| Morin et al,  2023 | 1 | 1 | 1 | 1 | 2 | 1 | 1 | 1 | 9 |
| Morin et al, 2019 | 1 | 1 | 1 | 1 | 2 | 1 | 0 | 1 | 8 |
| Ronaldson et al, 2021 | 1 | 1 | 1 | 1 | 2 | 1 | 1 | 1 | 9 |
| Triolo et al, 2024 | 1 | 1 | 1 | 1 | 2 | 1 | 1 | 1 | 9 |
| Valletta et al, 2021 | 1 | 1 | 1 | 1 | 2 | 1 | 1 | 1 | 9 |
| Wister et al, 2023 | 1 | 1 | 1 | 1 | 2 | 1 | 1 | 1 | 9 |
| Xiong et al, 2023 | 1 | 1 | 1 | 1 | 2 | 1 | 1 | 1 | 9 |
| Yao et al, 2020 | 1 | 1 | 1 | 1 | 2 | 1 | 1 | 1 | 9 |

# **Supplementary Table 5.** Conversion of colours coding charactering diseases for each multimorbidity patterns presented in the harvest plot (Figure 2).

| **First Author, Year** | **Identified Multimorbidity patterns** | **Modified Multimorbidity patterns included in the forest plot (Figure 2)** |
| --- | --- | --- |
| Bendayan et al,  2021 | 1. Heart Disease/Stroke 2. Asthma/Lung Disease 3. Arthritis/Hypertension 4. Depression/Arthritis 5. Hypertension/Cataracts/Diabetes 6. Psychiatric Problems/Depression 7. Cancer 8. Arthritis/Cataracts | 1. Cardiometabolic 2. Respiratory 3. MSK & Inflammatory 4. Neuropsych AND MSK & Inflammatory 5. Cardiometabolic AND Sensory 6. Neuropsych 7. Neoplasia 8. Sensory AND MSK & Inflammatory |
| Calvin et al,  2022 | Women:   1. Hypertension, diabetes & coronary heart disease 2. Pain, dyspepsia & depression 3. Cancer 4. Thyroid disorders 5. Pain, osteoporosis & dyspepsia 6. Asthma & COPD 7. Pain & hypertension   Men:   1. Hypertension, pain & dyspepsia 2. Pain, dyspepsia & prostate disorders 3. Coronary heart disease, hypertension & stroke 4. Asthma, COPD & psoriasis 5. Diabetes & hypertension 6. Cancer | Women:   1. Cardiometabolic 2. Neuropsych AND Pain 3. Neoplasia 4. Endocrine 5. MSK & Inflammatory AND Pain 6. Respiratory 7. Unspecific low comorbidity   Men:   1. Unspecific low comorbidity 2. Unspecific low comorbidity 3. Cardiometabolic 4. Respiratory 5. Cardiometabolic 6. Neoplasia |
| Gerrits et al, 2013 | 1. Cardiometabolic 2. Respiratory 3. Musculoskeletal 4. Digestive 5. Neurological 6. Endocrine 7. Cancer | 1. Cardiometabolic 2. Respiratory 3. MSK & Inflammatory 4. Gastrointestinal 5. Neuropsych 6. Endocrine 7. Neoplasia |
| Grande et al,  2021 | 1. Neuropsychiatric 2. Cardiovascular 3. Sensory impairment/cancer 4. Respiratory/metabolic/musculoskeletal | 1. Neuropsych 2. Cardiometabolic 3. Neoplasia AND Sensory 4. Respiratory AND MSK & Inflammatory |
| Ho et al,  2023 | 1. Cardiometabolic 2. Arthritis-cataract 3. Multimorbidity | 1. Cardiometabolic 2. MSK & Inflammatory AND Sensory 3. Complex high comorbidity |
| Hsu et al,  2013 | 1. Chronic respiratory disease 2. Cardiovascular disease 3. Gastrointestinal disease 4. Cancer 5. CRD + CVD 6. CRD + GI 7. CRD + cancer 8. CVD + GI 9. CVD + cancer 10. GI + cancer | 1. Respiratory 2. Cardiometabolic 3. Gastrointestinal 4. Neoplasia 5. Respiratory AND Cardiometabolic 6. Respiratory AND Gastrointestinal 7. Respiratory AND Neoplasia 8. Cardiometabolic AND Gastrointestinal 9. Cardiometabolic AND Neoplasia 10. Gastrointestinal AND Neoplasia |
| Hu et al,  2022 | 1. Obesity/other disorders 2. Cardio-cerebrovascular/respiratory/metabolic/musculoskeletal/depressive disorders 3. Tumor/genitourinary/digestive disorders | 1. Unspecific low comorbidity 2. Complex high comorbidity 3. Neoplasia AND Gastrointestinal |
| Li et al, 2023 | 1. Cardiometabolic 2. High comorbidity burden 3. Osteoarthrosis | 1. Cardiometabolic 2. Complex high comorbidity 3. MSK & Inflammatory |
| Khondoker et al,  2023 | 1. Mental health 2. Cardiometabolic 3. Inflammatory/autoimmune 4. Cancer-related pathophysiology | 1. Neuropsych 2. Cardiometabolic 3. MSK & Inflammatory 4. Neoplasia |
| Morin et al,  2023 | 1. Depression+Minimal Comorbidity 2. Depression + Medical Comorbidity 3. High Comorbidity | 1. Neuropsych AND Unspecific low comorbidity 2. Neuropsych AND Unspecific low comorbidity 3. Complex high comorbidity |
| Morin et al, 2019 | 1. Minimal Comorbidity 2. Chronic Pain-Osteoarthritis 3. Depression-Chronic Pain 4. Depression-Medical Comorbidity 5. High Comorbidity | 1. Unspecific low comorbidity 2. MSK & Inflammatory AND Pain 3. Neuropsych AND Pain 4. Unspecific low comorbidity 5. Complex high comorbidity |
| Ronaldson et al, 2021 | 1. Undefined multimorbidity 2. Cardiometabolic 3. Respiratory 4. Cardio/cerebrovascular 5. Reproductive 6. Pain/gastrointestinal | 1. Unspecific low comorbidity 2. Cardiometabolic 3. Respiratory 4. Cardiometabolic 5. Unspecific low comorbidity 6. Gastrointestinal AND Pain |
| Triolo et al, 2024 | 1. Unspecific 2. Metabolic 3. Sensory/anaemia 4. Thyroid/musculoskeletal 5. Cardiometabolic | 1. Unspecific low comorbidity 2. Endocrine 3. Sensory 4. Endocrine AND MSK & Inflammatory 5. Cardiometabolic |
| Valletta et al,  2021 | 1. Neuropsychiatric 2. Cardiovascular 3. Sensory impairment/cancer 4. Respiratory/metabolic/musculoskeletal 5. Unspecific | 1. Neuropsych 2. Cardiometabolic 3. Sensory AND Neoplasia 4. Respiratory AND MSK & Inflammatory 5. Unspecific low comorbidity |
| Wister et al, 2023 | 1. Cardio 2. Osteo 3. Respiratory 4. Cancer 5. Multiple 6. Other | 1. Cardiometabolic 2. MSK & Inflammatory 3. Respiratory 4. Neoplasia 5. Complex high comorbidity 6. Unspecific low comorbidity |
| Xiong et al, 2023 | 1. Relatively healthy pattern 2. Respiratory 3. Cardiovascular | 1. Unspecific low comorbidity 2. Respiratory 3. Cardiometabolic |
| Yao et al, 2020 | 1. Cardio-Metabolic 2. Respiratory 3. Arthritic-Digestive-Visual 4. Hepatic-Renal-Skeletal | 1. Cardiometabolic 2. Respiratory 3. Complex high comorbidity 4. Complex high comorbidity |
